# Diagnosis and Characterization of *Ditylenchus destructor* Isolated from *Mazus japonicus* in China

**DOI:** 10.3390/life13081758

**Published:** 2023-08-16

**Authors:** Wenwen Song, Mingming Dai, Qianqian Shi, Chen Liang, Fangmeng Duan, Honghai Zhao

**Affiliations:** Shandong Engineering Research Center for Environment-Friendly Agricultural Pest Management, College of Plant Health and Medicine, Qingdao Agricultural University, Qingdao 266109, China

**Keywords:** *Ditylenchus destructor*, *Mazus japonicus*, morphological characterization, molecular analysis

## Abstract

The potato rot nematode (*Ditylenchus destructor*) is one of the most destructive pests in the production of tuber crops, resulting in severely decreased yields and inferior product quality. In 2021, a great number of nematodes were detected in the roots of *Mazus japonicus*, a weed that is harmful to crop growth, in Qingdao, Shandong Province, China. The present study was undertaken to characterize and identify the nematodes isolated from *M. japonicus* through morphological identification and molecular approaches. Their morphological characteristics were highly consistent with the descriptions of *D. destructor* Thorne, 1945. The nematodes collected from *M. japonicus* were identified as *D. destructor* haplotype B using D1/D2 and sequence characterized amplified region (SCAR) primers. PCR-ITS-RFLP analysis was conducted to monitor intraspecific variations. In addition, the phylogenetic analysis of the internal transcribed spacer (ITS) demonstrated that this *D. destructor* population was clustered in haplotype B, supported by a 100% bootstrap value. Another assay, in which *M. japonicus* was inoculated with a mixture of the life stages of *D. destructor*, was performed. This assay showed that *M. japonicus* exhibited a high susceptibility to *D. destructor* in pots. This is the first record of *D. destructor* parasitizing *M. japonicus* in China, and it is of great importance because *M. japonicus* could be a potential reservoir for *D. destructor* in the field.

## 1. Introduction

The potato rot nematode (*Ditylenchus destructor* Thorne, 1945) is one of the most destructive pests of tuber crops [1]. *Ditylenchus destructor* was first found in potatoes (*Solanum tuberosum* L.) that displayed severely decreased yield and low quality [2,3]. The earliest record related to *D. destructor* can be traced back to Kuhn’s description of potato tuber rot disease in Germany in 1888, which was originally identified to have been caused by *D. dipsaci* [4]. In 1945, this pest was instead confirmed to be *D. destructor*, a new species different from *D. dipsaci* [4]. *Ditylenchus destructor* is a serious pest in a number of root and tuber crops, primarily in potatoes, sweet potatoes, and garlic. Due to economic losses, it has been listed as an internationally quarantined species. In China, *D. destructor* was first isolated in sweet potatoes (*Ipomoea batatas*) after causing severe damage to their production [5,6,7]. *Ditylenchus destructor* damages tubers, which results in tuber rot during storage. Similarly, the “dry rot” symptom caused by this nematode was first discovered in American ginseng (*Panax quinquefolium*) in China [8]. Additionally, *D. destructor* has always been a destructive pathogen to garlic (*Allium sativum*) in Japan [9,10,11]. Furthermore, due to an infestation of its own garlic with *D. destructor*, mainland Canada has implemented strict quarantine measures on infected fields to prevent its spread [12]. 

Biologically, the principal host of *D. destructor* is potatoes [13]. However, it can also infect other plants, such as alfalfa (*Medicago sativa*), red clover (*Trifolium pratense*), maize (*Zea mays*), and snap beans (*Phaseolus vulgaris*) [14,15,16]. Meanwhile, some weeds can also be its hosts, including *Cirsium arvense*, *Mentha arvensis*, *Potentilla anserine*, *Rumex acetosella,* and *Stachys palustris*, which have been identified as sources of *D. destructor* infection in some crops [17].

Previous studies have demonstrated that *D. destructor* has intraspecific variations among the different populations from different hosts or regions [18,19]. Two ITS types of *D. destructor* (haplotypes A and B) from twenty-one sweet potato populations with different geographical origins were first characterized by Wan et al. based on differences in their ITS sequences [20]. Furthermore, five additional haplotypes C, D, E, F, and G, isolated from *Astragalus membranaceus*, potatoes, and sweet potatoes, were distinguished on the basis of secondary ITS structures [21]. Recently, some novel haplotypes from Chinese medicinal herbs have been identified and named as haplotypes H, I, J, K, L, M, and N [13].

*Mazus japonicus* is another weed; it belongs to Scrophulariaceae and is distributed all over the world. It can produce a large amount of very small seeds, being a dominant species in weed seed banks [22]. Under natural conditions, this weed self-pollinates and can predominate without nitrogen fertilizer. As a result, its seed density has shown an increasing trend in recent years [23,24,25]. Nevertheless, in China, *M. japonicus* is used as a traditional Chinese medicine due to its functions of dissolving toxins, relieving pain, and invigorating the stomach. It is also applied for the topical treatment of malignant boils, pustulosis, and burns [26]. Similar findings have demonstrated that *M. japonicus* has excellent phytotoxic, antifungal, and cytotoxic potential, as therapeutically active novel saponins have been isolated from this plant [27]. However, up to now, there have been no reports of *D. destructor* parasitizing *M. japonicus*.

In May of 2021, large amounts of *M. japonicus* seedlings grew in Qingdao, Shandong Province, China. Although there was no obvious plant stunting or leaf wilting on the aboveground parts of the plants, a great number of nematodes were detected in their roots after staining with acid fuchsine. The objective of the present study was to conduct the identification and characterization of the nematodes isolated from *M. japonicus* through morphological, morphometric, and molecular analysis.

## 2. Materials and Methods

### 2.1. Nematodes Isolation

Infected roots of *M. japonicus* were collected from Jiaozhou Qingdao, China, in May of 2021. A *D. destructor* population from potatoes was obtained from Pingdu, Qingdao, China. Root samples of *M. japonicus* were washed with sterile water and surface-sterilized in 75% ethanol for 30 s, followed by washing in sterile water three times. Afterward, some of the samples were used to evaluate nematode invasion by staining the infected roots using acid fuchsine (Aladdin, Shanghai, China) [28]. To obtain pure cultures of this population for further analysis, the plants were cut into small pieces and incubated in sterile water at 23 °C for 24 h. Subsequently, individual nematodes were isolated with a Baermann funnel and transferred to fungal cultures (*Fusarium solani*) for propagation [13,29].

### 2.2. Morphological Measurements

A nematode suspension was incubated at 60 °C for 10 min and then cooled down to room temperature. The same volume of 5% formalin was added to fix the nematodes. A total of 20 females and 20 males were randomly selected for morphological measurements under an Axioscope 5 microscope (Zeiss, Jena, Germany) using Zeiss 3.0 software. The De Man formula was employed to calculate morphological features.

### 2.3. Molecular Identification Using Universal Primers and Specific Primers

DNA was extracted from mature female nematodes according to the method described by Wang et al. [30]. Two pairs of primers were used to amplify the ITS region of the ribosomal DNA; one pair of universal primers, TW81/AB28, has been described by Maafi et al. [31]. Molecular identification of the species was conducted with the other pair of D1/D2 primers, which are specific to *D. destructor* [32]. Additionally, another two pairs of species-specific primers for the SCARDdS1/DdS2 and DdL1/DdL2 [20] were used to determine which nematode haplotype was parasitizing *M. japonicus.*

The PCR reaction was carried out in a 25 µL reaction mixture containing 12.5 µL of 2×PCR Taq Mix (Takara, Dalian, China), 2 µL of a DNA template, 2 µL of each primer (10 μM), and 6.5 µL of ddH_2_O. The reaction parameters were set as follows: 95 °C for 5 min, followed by 35 cycles each at 95 °C for 30 s, 60 °C for 10 s, and 72 °C for 30 s, with a final extension stage at 72 °C for 5 min. The PCR products were analyzed with gel electrophoresis and then sequenced by Tsingke Biological Technology Company Limited (Qingdao, China). The ITS and D1D2 product sequences were submitted to the GenBank database to obtain the corresponding accession numbers. 

### 2.4. RFLP Analysis of ITS Region

To assess the variability within the different populations, amplified rDNA-ITS products from populations of nematodes from *M. japonicus* and *D. destructor* from potatoes were digested separately with each of the restriction enzymes, respectively. The available restriction enzymes for RFLP analysis were screened on the basis of the sequences of these accessions using SnapGene 7 software. Digestions were performed following the manufacturer’s recommendations. Briefly, the ITS products were digested by their respective restriction enzymes. Each digestion reaction had 2 μL of 10× reaction buffer, 1 μL of restriction enzyme, 10 μL of ITS product, and 7 μL of ddH_2_O. The reaction mixture was incubated at 37 °C for 1.5 h. Afterward, restriction products were separated on 3% agarose gels, stained with ethidium bromide, visualized on a UV transilluminator (Bio-Rad, Hercules, CA, USA), and photographed.

### 2.5. Phylogenetic Analysis

The ITS sequences of *Ditylenchus* species obtained from GenBank were used to construct a phylogenetic tree. The sequences of *D. myceliophagus* (AM232236, DQ151458) were used as the outgroup taxon for analysis of ITS-rDNA (Table 1). Nucleotide sequences were aligned with other sequences in GenBank using ClustalX v1.83 software. The phylogenetic tree was constructed with the maximum likelihood (ML) method and MEGA 11 software using 1000 replicates. DNAMAN 8.0 software was used for sequence alignment.

### 2.6. Host Reaction of M. japonicus to Ditylenchus destructor

The nematodes isolated from *M. japonicus* were cultured on a PDA medium inoculated with *F. solani* at 23 °C. After 30 days of incubation, nematodes at mixed life stages were washed off with sterile water and collected using the Baermann funnel [29]. The nematodes were transferred into 1.5 mL Eppendorf tubes, diluted with sterile water to 1 mL, and mixed thoroughly.

The pathogenicity of the nematodes from *M. japonicus* was examined in potatoes as well as *M. japonicus*. *M. japonicus* seeds were surface-sterilized in 5% sodium hypochlorite and then washed three times with sterile water. Afterward, they were left to germinate for 7 days on germination paper at 23 °C and in the dark. Each healthy seedling was then transplanted into a pot (7 cm diameter × 8 cm deep) filled with sterilized sandy loam. Meanwhile, control infection tests were conducted on the potato cultivar Helan 15, as has been described by Li et al. but with minor modifications [33]. Briefly, Helan 15 plants were grown in a pot filled with 500 g of sandy soil. Each seeding was maintained in a growth chamber under a 16 h light/8 h dark photoperiod, at 23 °C and with 60% relative humidity. The nematodes were inoculated onto the potato roots after 2 weeks and onto the *M. japonicus* after 2 months. A total of 2 mL of a suspension containing approximately 1000 mixed-stage nematodes collected from *M. japonicus* was inoculated into the soil around the roots of each plant. All of the seedlings were incubated at 23 °C under a 16 h photoperiod. After 60 days post inoculation, all of the plants were uprooted and their roots stained using acid fuchsine [28]. Potato cubes were cut into small pieces, and nematodes were isolated using the Baermann funnel. After 24 h, the nematodes were observed under a microscope and counted. This experiment was conducted three times. Infection of different hosts with the nematode population from *M. japonicus* was determined with statistical analysis using SPSS 20 software.

The reproduction factor, (RF) = Pf/Pi (Pf is the final population and Pi is the initial population), was determined.

## 3. Results

### 3.1. Nematode Observation in Mazus japonicus

**Figure 1:**
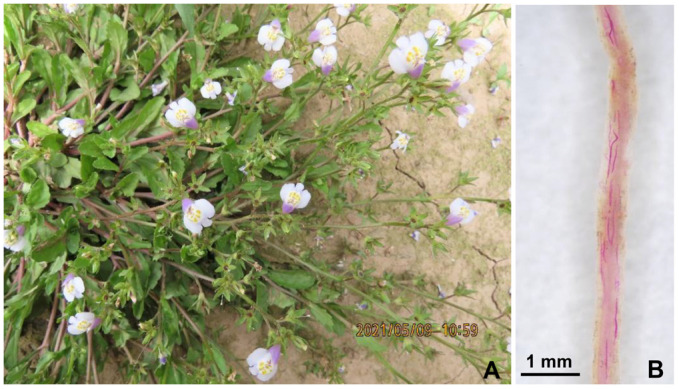
Identification of nematodes infecting *Mazus japonicus*. The aboveground part of *M. japonicus* in the field (**A**). The infected roots of *M. japonicus* after staining with acid fuchsine (**B**).

As shown in Figure 1, there were no obvious symptoms, such as plant stunting or leaf wilting, on the aboveground part of the *M. japonicus* (Figure 1A). However, a great number of nematodes were observed in the *M. japonicus* roots after the staining with acid fuchsine (Figure 1B), with densities ranging from 526 to 1158 nematodes per gram of fresh roots.

### 3.2. Morphological Characteristics

**Figure 2:**
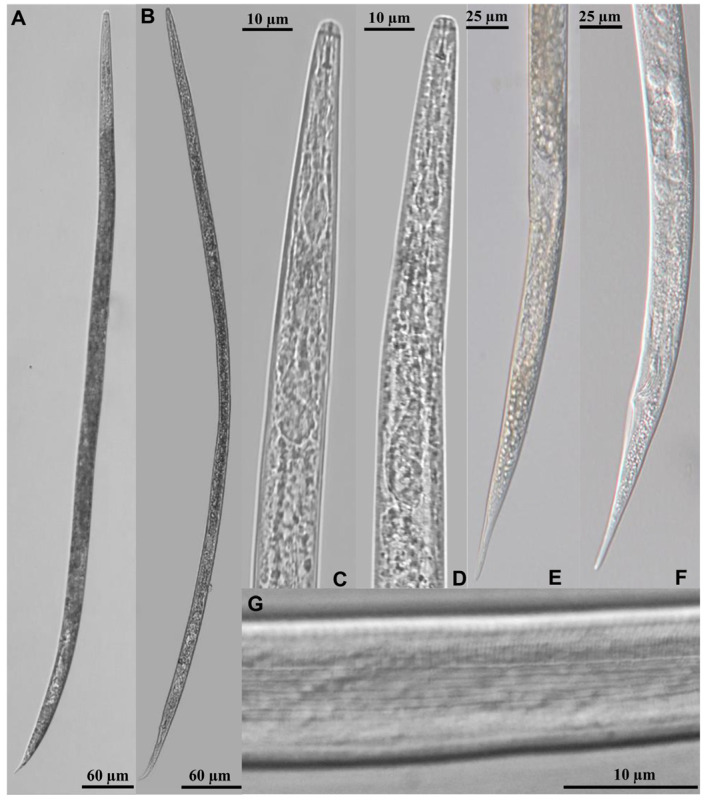
Micrographs of nematode population extracted from *Mazus japonicus* in China. Body length of female (**A**) and male (**B**); anterior region of female (**C**) and male (**D**); vulva and tail of female (**E**); spicules and tail of male (**F**); and lateral field of female (**G**).

The morphological measurements of the nematode females (*n =* 20) and males (*n =* 20) are shown in Table 2. Most morphometric data were consistent with the description of *D. destructor* Thorne, 1945 [4,34,35], with the exception of stylet length (ST). The ST of the *Mazus japonicus* population was shorter than that of *D. destructor* Thorne, 1945. Photographs of the morphologies of the whole bodies of the typical female and male nematodes were taken (Figure 2A,B). The stylets of both the females and the males were small and thin (Figure 2C,D). In the females, each vulva was slightly protruded; some even protracted to the esophagus area. Each posterior uterus extended approximately three-quarters of the distance to the anus. The tails of the females were conical, with finely rounded termini (Figure 2C,E). The male tail terminus was thin and round, and the spicules bent slightly to the ventral surface. The bursa began at the opposite position of the front end of the spicules, extending backward to 1/4 of the length of the tail (Figure 2D,F). The lateral field of each female had six incisures (Figure 2G).

### 3.3. Amplification of ITS and Specific Region

A molecular determination was conducted to evaluate the molecular features of the nematodes isolated from the *M. japonicus* roots for further species identification. Nucleotide fragments of the ITS regions from *D. destructor* of the potato population and the *M. japonicus* population were amplified, sequenced, and deposited in GenBank, with accessions numbers ON753817 and OL677340, respectively. 

**Figure 3:**
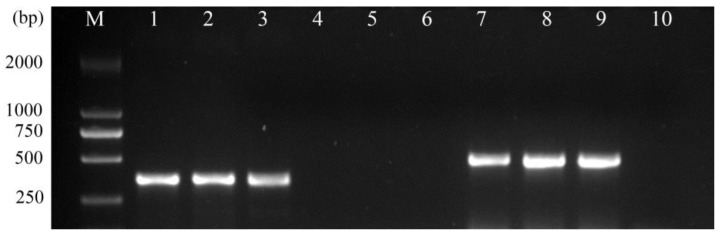
Amplification of ITS and specific region of nematodes isolated from *M. japonicus*. M: DL2000 marker; 1–3: Amplification using D1/D2 primers; 4–6: Amplification using DdS1/DdS2 primers; 7–9: Amplification using DdL1/DdL2 primers; 10: Water served as the negative control.

**Figure 4:**
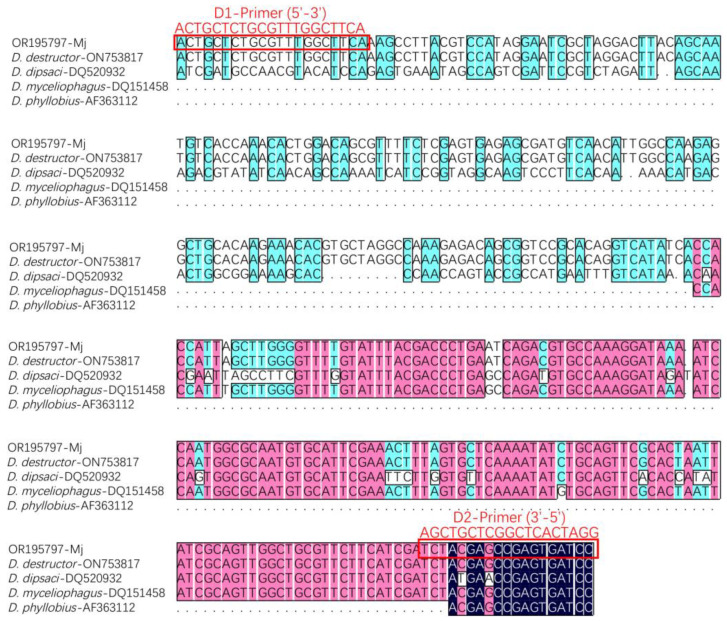
Alignment of D1–D2 sequences for *Ditylenchus*.

The 346 bp fragments, amplified by primers D1/D2, were observed under UV light, with no fragments amplified by DdS1/DdS2, respectively (Figure 3). This demonstrated that the nematodes extracted from the *M. japonicus* roots were *D. destructor*. The D1–D2 sequence of the *M. japonicus* population was submitted to GenBank under accession number OR195797. Furthermore, a genetic comparison of the D1–D2 sequence was performed using DNAMAN 8.0 software. As shown in Figure 4, this comparison (OR195797) showed 100% identity, with the corresponding gene sequence of *D. destructor* published in GenBank (ON753817). As expected, there were obvious differences between the D1–D2 sequences of *D. destructor* (OR195797 and ON753817) and the other intragenus species of *Ditylenchus*, including *D. dipsaci*, *D. myceliophagus,* and *D. phyllobius*. 

Additionally, a 485 bp fragment of the *M. japonicus* population was amplified with the specific primers DdL1 and DdL2 (Figure 3), which demonstrated that these nematodes belonged to *D. destructor* haplotype B.

### 3.4. RFLP Analysis of ITS Region

**Figure 5:**
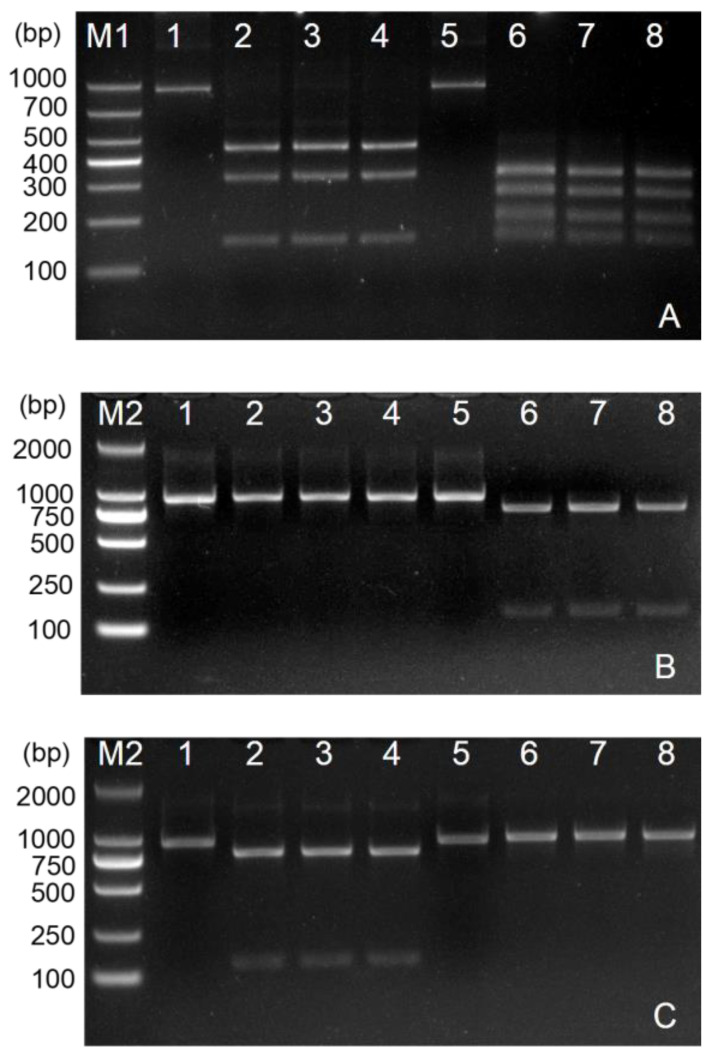
Electropherogram of the RFLP assay on the two populations of *Ditylenchus destructor* from *M. japonicus* and from potatoes. Digestion of the ITS amplicon with BsiHKAI (**A**), BstAPI (**B**), and FspI (**C**). M1: DL1000 marker; M2: DL2000 marker; Lane 1: The ITS amplicon of the potato population; Lanes 2–4: Digestion of the ITS amplicon of the potato population with restriction enzymes; Lane 5: The ITS amplicon of the *Mazus japonicus* population; Lanes 6–8: Digestion of the ITS amplicon of the *M. japonicus* population with restriction enzymes.

The restriction enzymes BsiHKAI, BstAPI, and FspI were determined according to the sequence differences at positions 256, 154, and 137, respectively, of the base pairs. Thus, all three of these enzymes were used for the RFLP analysis. As shown in Figure 5A, BsiHKAI digestion resulted in four bands (256/191/149/319) in the ITS sequence of the *M. japonicus* population, and three bands (447/149/319) were produced for the potato population; BstAPI could digest the ITS product from the *M. japonicus* population only. In detail, BstAPI could not digest the *D. destructor* ITS products from the potatoes due to there being no recognition site on the ITS sequence for this population (Lanes 2–4, Figure 5B), whereas the ITS amplicon from the *M. japonicus* population could be digested by BstAPI into two fragments (154/761), which are shown in Lanes 6–8 of Figure 5B. FspI could digest only the *D. destructor* ITS product collected from the potatoes (Figure 5C). Two fragments (137/778) were generated by the FspI digestion of these ITS products from the potato population (Lanes 2–4, Figure 5C). The ITS products from the *M. japonicus* population could not be cleaved by FspI (Lanes 6–8, Figure 5C). Taken together, the electrophoretic profiles of the restriction fragments from the two populations were fully consistent with the theoretical expectations. BsiHKAI, BstAPI, and FspI were able to identify and clearly discriminate *D. destructor* from the *M. japonicus* population and the potato population.

### 3.5. Phylogenetic Analysis

**Figure 6:**
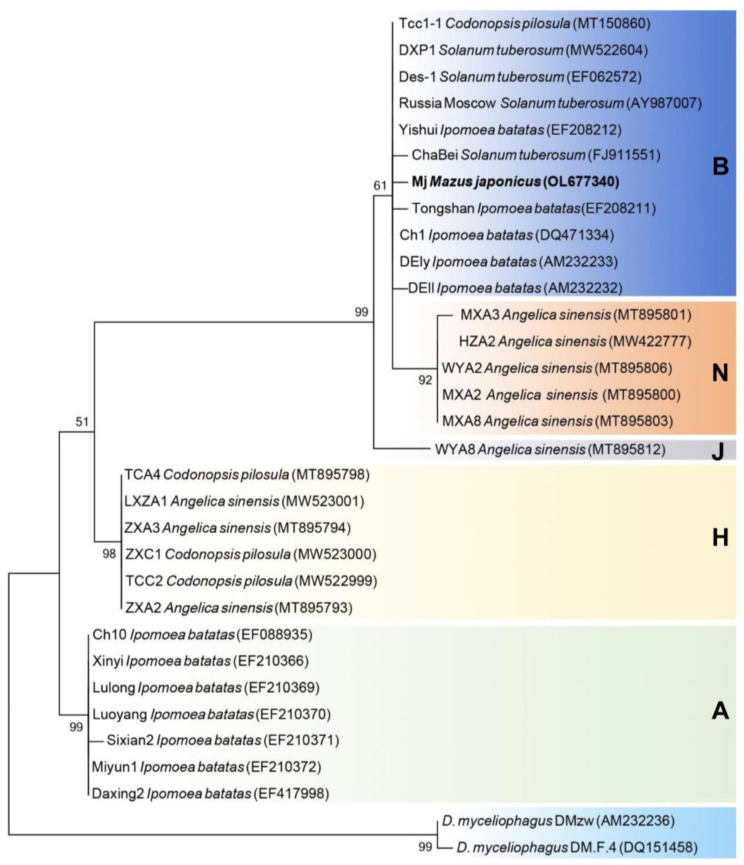
Phylogenetic tree inferred from a maximum likelihood method based on the ITS genes of *Ditylenchus* isolates from GenBank. Two isolates of *D. myceliophagus* were used as the outgroup.

To verify the reliability of these results based on morphological characteristics and molecular identification, a phylogenetic analysis of the ITS region was performed using ML with 1000 replicates and MEGA 11 software. The nucleotide sequences of 31 isolates (*Ditylenchus* species) from the NCBI GenBank were downloaded to construct a phylogenetic tree. As shown in Figure 6, five haplotypes of *D. destructor* were distinguished in this study. The *Mazus japonicus* population Mj belonged to haplotype B. Similarly, the population Tcc-1, which was isolated from the Chinese medicinal herb *Codonopsis pilosula*, was also identified as haplotype B. In addition, haplotype B was also found in *I. batatas* and *S. tuberosum*, but not in the Chinese medicinal herb *Angelica sinensis*. In contrast, haplotypes J and N were found only in *A. sinensis*. Haplotype H was found in both *A. sinensis* and *C. pilosula*, and haplotype A was found only in *I. batata*.

### 3.6. Host Reaction of M. japonicus to D. destructor

**Figure 7:**
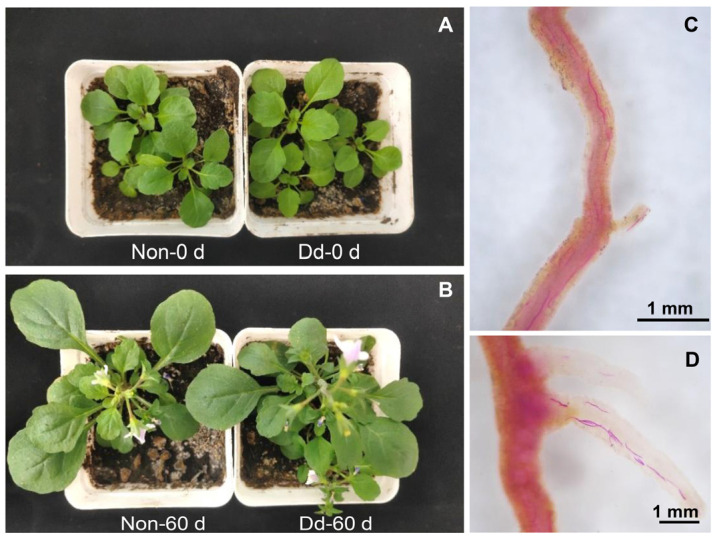
The aboveground part and roots of *Mazus japonicus* before and after inoculation of *Ditylenchus destructor*. The aboveground part of *M. japonicus* before inoculation of *D. destructor* (**A**). The aboveground part of *M. japonicus* at 60 days post-inoculation of *D. destructor* (**B**). The main root (**C**) and lateral roots (**D**) of *M. japonicus* at 60 days post-inoculation of *D. destructor*. Non: No nematodes inoculation; Dd: *D. destructor* inoculation.

The infection and reproduction of the nematodes in the *M. japonicus* were evaluated. As shown in Figure 7A,B, before and after inoculation, there were no obvious symptoms of infection with the nematodes compared with the non-inoculated plant. However, staining revealed mixed-stage nematodes in the dissected roots, with population densities ranging from 366 to 1043 nematodes per gram of fresh roots (Figure 7C,D). 

As shown in Table 3, there was no significant difference between the number of nematodes in *M. japonicus* and that in the potatoes. However, a dramatic reduction was observed in the number of nematodes in the soil of the *M. japonicus* compared with that in the soil where the potatoes were grown. A slight decrease in the RF was detected in the *M. japonicus* in comparison with that in potatoes: 2.68 and 3.18, respectively. These results demonstrate that *M. japonicus* shows a high susceptibility to *D. destructor.*

## 4. Discussion

*Ditylenchus destructor* can infect more than 100 host plant species [21]. However, its damage exhibits different characteristics among the different plants [36]. In general, it causes common symptoms, such as discoloration and rotting in tubers, stolons, bulbs, rhizomes, and roots. However, it rarely attacks the aboveground part of any plant, with the exception of the base of the stem. Therefore, aboveground symptoms, such as dwarfing or curling and discoloration of leaves, have occurred seldomly [17]. Similar results were observed in this study. No distinct symptoms were observed in the aboveground parts of the *M. japonicus* infected with the *D. destructor*. Additionally, there were no obvious symptoms in the roots. Consequently, it was difficult to identify this infection from only the visible symptoms, which implies that the *D. destructor* that parasitized the *M. japonicus* exhibits a high concealment. This work, being the first such report for China, shows, for the first time, that *M. japonicus* is a novel host of *D. destructor*. It also demonstrates the great importance of the integrated management of nematodes in agricultural fields and the important crops, like potatoes, that can be hosts of these nematodes.

The present study indicates a minor variation in stylet length (ST) compared with previous descriptions of *D. destructor* Thorne, 1945, which might be caused by different host plants. Similar findings have observed that host plants could influence some minor variations in characters [34,35]. In addition, another population isolated from potatoes in China was identified as *D. destructor* Thorne, 1945, with an ST of 5.7~8.0 μm in females and 6.3~8.0 μm in males [37], which was slightly shorter than that of the *M. japonicus* population but much shorter than that of the potato populations of *D. destructor* Thorne, 1945 derived from Canada, Iran, and the USA [34,35,38]. Therefore, the ST of *D. destructor* varies regionally, which may be influenced by host species and/or climate.

In the present work, the *D. destructor* population that infects *M. japonicus* is identified as haplotype B. Haplotype B was also discovered in *C. pilosula* but not in *A. sinensis* [13]. The *D. destructor* population isolated from *A. membranaceus* belonged to haplotype F [13,21]. These results indicate different haplotypes among the different *D. destructor* populations isolated from Chinese medicinal herbs. In addition, haplotype B could also be observed in both potatoes and sweet potatoes in China, Russia, and Europe. However, haplotype A was predominant only in sweet potatoes found in China, whereas haplotype G was found only in potatoes in Russia and Europe [21]. These findings demonstrate that distribution patterns and host plants vary diversely among the different haplotypes. Although the *D. destructor* populations from *M. japonicus* and potatoes belonged to haplotype B, a significant variation was observed in the ITS-rDNA sequence through ITS-RFLP analysis, implying that *D. destructor* has intraspecific variation among different populations from different hosts or regions. Similar findings can be observed in previous studies [39,40].

In China, most *D. destructor* populations can be divided into two haplotypes, A and B [41], which can be distinguished at the morphological and molecular levels. In terms of morphology, the body lengths of both the males and females of haplotype B are significantly smaller than those of haplotype A. The V values of the females of haplotype B are lower than those of haplotype A, but the A values of haplotype B are higher than those of haplotype A. In addition, a slight difference exists between the B and C values [42]. At the molecular level, these two haplotypes can be distinguished with specific ITS primers due to the lack of 188 bp fragments of haplotype A. Genetic differences might result in differences in morphological characters between the two haplotypes. However, haplotypes A and B cannot be distinguished based on pathogenicity. The pathogenicities of different cases of the same haplotype for the same plant host can vary significantly [33]. Therefore, morphological characteristics, molecular identification, and pathogenicities among different populations in different hosts or regions should be systematically analyzed to improve the detection and classification of *D. destructor.*

Our results have shown that *M. japonicus* has a high susceptibility to *D. destructor*. It is worth noting that a significant difference was observed between the *D. destructor* numbers in the soils of *M. japonicus* and potatoes. As *D. destructor* can also feed on fungi, different species of fungi might exist in the rhizosphere soils of the different host plants. In addition, root exudates of different host plants might affect the *D. destructor* numbers in soil. Similar findings were obtained for *Heterodera glycines* that was attracted to soybeans but unattracted to either marigolds or peppers [43]. Further studies need to be performed to find out which individual factors influence the numbers of nematodes in soil.

In summary, *D. destructor* occurs in 12 provinces, posing a serious threat to potato production in China [44]. This study has confirmed that *M. japonicus* shows a high susceptibility to *D. destructor* haplotype B under natural conditions. To our knowledge, this is the first report of *D. destructor* infestation of *M. japonicus* in China and in the world. This finding is very important because *M. japonicus* can be a potential reservoir for *D. destructor* in potato fields or elsewhere. This has provided valuable information for the management of this weed in crop production.

## 5. Conclusions

In this study, we confirmed, based on morphological characteristics, molecular identification, and pathogenicity tests, that the causal pest in *M. japonicus* roots is *D. destructor*. Additionally, *M. japonicus* showed a high susceptibility to *D. destructor*. To our knowledge, this is the first report that *M. japonicus* can be infected with *D. destructor* under natural conditions, which should attract attention because *M. japonicus* can be a potential reservoir for *D. destructor* in potato and other fields. This is of great significance for the integrated management of *D. destructor* and *M. japonicus*.

## Figures and Tables

**Table 1:** List of isolates, hosts, and GenBank accession numbers of *Ditylenchus* species used.

Species	Isolate	Host	GenBank Accession Number
*Ditylenchus destructor*	DEll	*Ipomoea batatas*	AM232232
	DEly	*Ipomoea batatas*	AM232233
	Ch1	*Ipomoea batatas*	DQ471334
	Ch10	*Ipomoea batatas*	EF088935
	Tongshan	*Ipomoea batatas*	EF208211
	Yishui	*Ipomoea batatas*	EF208212
	Xinyi	*Ipomoea batatas*	EF210366
	Lulong	*Ipomoea batatas*	EF210369
	Luoyang	*Ipomoea batatas*	EF210370
	Sixian2	*Ipomoea batatas*	EF210371
	Miyun1	*Ipomoea batatas*	EF210372
	Daxing2	*Ipomoea batatas*	EF417998
	Russia Moscow	*Solanum tuberosum*	AY987007
	Des-1	*Solanum tuberosum*	EF062572
	ChaBei	*Solanum tuberosum*	FJ911551
	DXP1	*Solanum tuberosum*	MW522604
	WYA8	*Angelica sinensis*	MT895812
	LXZA1	*Angelica sinensis*	MW523001
	HZA2	*Angelica sinensis*	MW422777
	ZXA3	*Angelica sinensis*	MT895794
	WYA2	*Angelica sinensis*	MT895806
	MXA2	*Angelica sinensis*	MT895800
	MXA8	*Angelica sinensis*	MT895803
	MXA3	*Angelica sinensis*	MT895801
	ZXA2	*Angelica sinensis*	MT895793
	TCA4	*Codonopsis pilosula*	MT895798
	Tcc1-1	*Codonopsis pilosula*	MT150860
	ZXC1	*Codonopsis pilosula*	MW523000
	TCC2	*Codonopsis pilosula*	MW522999
*Ditylenchus myceliophagus*	DMzw	*Agaricus bisporus*	AM232236
	DM.F.4	*Agaricus bisporus*	DQ151458

**Table 2:** Morphological measurements of females and males (µm).

Population\Character	*Mazus japonicus* Population		*Ditylenchus destructor* Thorne, 1945 [4]		*Ditylenchus destructor* Thorne, 1945 [34]		*Ditylenchus destructor* Thorne, 1945 [35]	
	Female (*n* = 20)	Male (*n* = 20)	Female	Male	Female (*n =* 80)	Male (*n =* 40)	Female (*n =* 489)	Male (*n =* 422)
L	986.88 ± 113.27 (833.92~1145.36)	993.63 ± 22.85 (965.58~1028.25)	800~1400	800~1300	925~1396	929~1232	994 (603~1468)	894 (600~1254)
W	28.91 ± 4.99 (22.62~41.43)	23.3 ± 0.74 (22.68~24.74)	-	-	28–43	26–33	-	-
a	34.57 ± 3.57 (27.3~39.57)	42.48 ± 1.48 (40.3~44.48)	30~35	34~40	26.27-39.40	32.10~41.29	36.6 (21.4~52.2)	42.2 (27.1~59.5)
DGO	1.18 ± 0.09 (1.06~1.33)	1.18 ± 0.1 (1.07~1.31)			-	-	-	-
ST	8.14 ± 0.36 (7.47~8.54)	7.42 ± 0.43 (6.86~7.95)	-	-	10.5~11.5	10.2–11.8	10.3 (8~13)	10.1 (8.5~12)
AM	58.34 ± 5.07 (49.27~66.89)	53.30 ± 2.07 (49.4~55.49)			-	-	-	-
b	8.54 ± 0.55 (7.63~9.23)	7.35 ± 0.42 (6.99~8.06)	8~10	7~8	7.81~10.6	6.30~9.40	7.4 (4.9~11.7)	6.8 (4.8~9.0)
Tail	74.74 ± 7.89 (63.92~89.76)	71.42 ± 4.34 (66.47~76.98)	-	73–80	68–98	51.38~73.15	67.3 (39.5~90.5)	63.3 (45~87.5)
c	13.23 ± 0.96 (11.5~14.43)	13.7 ± 0.49 (13.2~14.48)	15~20	12~16	11.67~17.21	12.69~16.29	14.8 (11.4~27.8)	14.1 (11.0~19.1)
ABW	15.92 ± 2.54 (12.43~20.92)	14.5 ± 1.05 (12.59~15.71)	-	-	-	-	-	-
c’	4.74 ± 0.40 (4.18~5.82)	4.94 ± 0.39 (4.25~5.33)	-	-	-	-	4.3 (2.6~7.2)	5.0 (3.1~6.9)
PUS	109.83 ± 3.46 (101.93~115.14)	-	-	-	-	-	-	-
V.a.	126.93 ± 16.68 (110.21~152.15)	-	-	-	-	-	-	-
V	80.62 ± 0.90 (79.16~81.54)	-	78~83	-	76.60~83.61	-	80.8 (77.1~84.9)	-
SPI	-	21.71 ± 1.68 (19.79~23.99)	-	-	-	-	-	22.0 (18~28)
Bur	-	47.25 ± 5.02 (38.52~52.49)	-	-	-	-	-	-

Note: Abbreviations of morphological features. L: Body length; W: Greatest body width; a: Body length divided by greatest body width; DGO: Dorsal gland orifice to stylet; ST: Stylet length; AM: Distance from anterior end to center of median esophageal bulb valve; b: Body length divided by esophageal length; Tail: Tail length; c: Body length divided by tail length; ABW: Anal body width; c’: Tail length divided by body width at anus; PUS: Post uterine sac; V.a.: Distance from vulva to anus; V: Distance from head end to vulva ×100 divided by body length; SPI: Spicules length; Bur: Bursa.

**Table 3:** Host reaction of *Mazus japonicus* to *Ditylenchus destructor*.

Host	Number of Nematodes in the Plants	Number of Nematodes in the Soil (per Pot)	Reproduction Factor (RF)
*Solanum tuberosum*	3092.40 ± 162.53	86.42 ± 5.64	3.18 ± 0.16
*Mazus japonicus*	2671.40 ± 223.56	9.50 ± 1.00 *	2.68 ± 0.22

Note: Data are shown with mean and standard error. Significantly different numbers of nematodes in soil of *Mazus japonicus* compared with that of *Solanum tuberosum* (control) were determined: *, *p* < 0.05, according to Student’s *t*-test.

## Data Availability

Sequencing data reported in this study were deposited to GenBank, available online at https://www.ncbi.nlm.nih.gov/genbank/ (accessed on 5 December 2021 (OL677340), 19 June 2022 (ON753817), and 3 July 2023 (OR195797)).

## References

[1] Liu B. (2006). Morphology and Specific Detection of Some Populations of *Ditylenchus destructor* Occurring in China. Ph.D. Thesis.

[2] Liu X.B., Ge J.J., Tan Z.Q., Cao A.X. (2006). First report of *Ditylenchus destructor* Thorne, 1945 infecting potato in China. Plant Prot..

[3] Ou S.Q., Wang Y.W., Peng D.L., Qiu H., Bai Q.R., Shi S.S. (2017). Discovery of potato rot nematode, *Ditylenchus destructor*, infesting potato in inner Mongolia, China. Plant Dis..

[4] Thorne G. (1945). *Ditylenchus destructor*, n. sp., the potato rot nematode, and *Ditylenchus dipsaci* (Kühn, 1857) Filipjev, 1936, the teasel nematode (Nematoda: Tylenchidae). Proc. Helminthol. Soc. Wash..

[5] Guo X.D., Xie Y.Z., Jia Z.D., Ma P.Y., Bian X.F. (2012). Study on sweet potato stem nematode disease. Plant Dis. Pests.

[6] Zhang C.L., Sun H.J., Xu Z., Yang D.J., Zhao Y.Q., Xie Y.P. (2015). Attractant and repellent effects of sweet potato root exudates on the potato rot nematode, *Ditylenchus destructor*. Nematology.

[7] Fan W.J., Wei Z.R., Zhang M., Ma P.Y., Liu G.L., Zheng J.L., Guo X.D., Zhang P. (2015). Resistance to *Ditylenchus destructor* infection in sweet potato by the expression of small interfering RNAs targeting *unc*-15, a movement-related gene. Phytopathology.

[8] Zhang G.Z., Zhang H.W. (2007). First report of root rot of American ginseng (*Panax quinquefolium*) caused by *Ditylenchus destructor* in China. Plant Dis..

[9] Fujimura T., Washio S., Nishizawa T. (1986). Garlic as a new host of the potato–rot nematode, *Ditylenchus destructor* Thorne. Jpn. J. Nematol..

[10] Fujimura T., Ichita T., Kimura T. (1989). Occurrence of potato-rot nematode, *Ditylenchus destructor* Thorne, in garlic and control.1. Evaluation of treatments applied before planting and after harvest for control. Jpn. J. Nematol..

[11] Cheng Z.J., Toyota K., Aoyama R. (2019). Relationship among the potato rot nematode, *Ditylenchus destructor*, densities in soil, root and garlic (*Allium sativum*) bulbs, and rot damage in stored garlic bulbs. Nematology.

[12] Yu Q., Zaida M.A., Hughes B., Celetti M. (2012). Discovery of potato rot nematode, *Ditylenchus destructor*, infesting garlic in Ontario, Canada. Plant Dis..

[13] Ni C.H., Han B., Liu Y.G., Maria M., Liu S.M., Li W.H., Shi M.M., Li H.X., Peng D.L. (2023). Diagnosis and characterization of the ribosomal DNA-ITS of potato rot nematode (*Ditylenchus destructor*) populations from Chinese medicinal herbs. J. Integr. Agric..

[14] Darling H.M., Adams J., Norgren R.L. (1983). Field eradication of the potato rot nematode, *Ditylenchus destructor*: A 29-year history. Plant Dis..

[15] Macguidwin A.E., Slack S.A. (1991). Suitability of alfalfa, corn, oat, red clover, and snapbean as hosts for the potato rot nematode, *Ditylenchus destructor*. Plant Dis..

[16] Pan F.J., Li F., Mao Y.Z., Liu D., Chen A.S., Zhao D., Hu Y.F. (2021). First Detection of *Ditylenchus destructor* parasitizing maize in northeast China. Life.

[17] EPPO/OEPP (2017). PM 7/87 (2) *Ditylenchus destructor* and *Ditylenchus dipsaci*. Bull. OEPP/EPPO Bull..

[18] Lagisz M., Poulin R., Nakagawa S. (2013). You are where you live: Parasitic nematode mitochondrial genome size is associated with the thermal environment generated by hosts. J. Evol. Biol..

[19] Andriuzzi W.S., Wall D.H. (2018). Grazing and resource availability control soil nematode body size and abundance–mass relationship in semi-arid grassland. J. Anim. Ecol..

[20] Wan F., Peng D.L., Yang Y.W., He Y.Q. (2008). Species specific molecular diagnosis of *Ditylenchus destructor* populations occurring in China. Acta Phytopathol. Sin..

[21] Subbotin S.A., Deimi A.M., Zheng J.W., Chizhov V.N. (2011). Length variation and repetitive sequences of Internal Transcribed Spacer of ribosomal RNA gene, diagnostics and relationships of populations of potato rot nematode, *Ditylenchus destructor* Thorne, 1945 (Tylenchida, Anguinidae). Nematology.

[22] Zhang C.B., Ma B., Qiang S. (2012). Analyses of species composition and diversity of weed seed bank of main crop fields in Jiangsu Province and its correlation with environmental factors. J. Plant Resour. Environ..

[23] Li S.S., Wei S.H., Zuo R.L., Wei J.G., Qiang S. (2012). Changes in the weed seed bank over 9 consecutive years of rice-duck farming. Crop Prot..

[24] Mikio K. (1978). Comparative Studies on the reproductive systems of *Mazus japonicus* and *M. mlquelli* (Scrophulariaceae). Plant Syst. Evol..

[25] Mi W.H., Gao Q., Sun Y., Zhao H.T., Yang X., Guo X.G., Chen J.Q., Wu L.H. (2018). Changes in weed community with different types of nitrogen fertilizers during the fallow season. Crop Prot..

[26] Wang G.Q. (2014). National Chinese Herbal Medicine Compilation.

[27] Farooq U. (2013). Biological Evaluation of the plant *Mazus japonicus* Albiflorus (Scrophulariaceae). Res. Rev. J. Pharmacogn. Phytochem..

[28] Daykin M., Hussey R., Barker K.R., Carter C.C., Sasser J.N. (1985). Staining and histopathological techniques in nematology. An Advanced Treatise on Meloidogyne: Methodology.

[29] Viglierchio D.R., Schmitt R.V. (1983). On the methodology of nematode extraction from field samples: Baermann funnel modifications. J. Nematol..

[30] Wang J.L., Zhang J.C., Gu J.F. (2011). Method of extract DNA from a single nematode. Plant Quar..

[31] Maafi Z.T., Subbotin S.A., Moens M. (2003). Molecular identification of cyst-forming nematodes (*Heteroderidae*) from Iran and a phylogeny based on ITS-rDNA sequences. Nematology.

[32] Liu B., Mei Y., Zheng J. (2007). Species-specific detection of inter-populations of *Ditylenchus destructor*. J. Zhejiang Univ. Agric. Life Sci..

[33] Li Y.Q., Huang L.Q., Jiang R., Han S.M., Chang Q., Li Y.M., Chen Z.J., Peng H., Huang W.K., Guo J.M. (2022). Molecular characterization of internal transcribed spacer (ITS) of ribosomal RNA gene, haplotypes and pathogenicity of potato rot nematode *Ditylenchus destructor* in China. Phytopathol. Res..

[34] Wu L.Y. (1960). Comparative study of *Ditylenchus destructor* Thorne, 1945 (Nematoda: Tylenchidae), from potato, bulbous Iris, and dahlia, with a discussion of de Man’s ratios. Can. J. Zool..

[35] Hashemi K., Karegar A. (2019). Description of *Ditylenchus paraparvus* n. sp. from Iran with an updated list of *Ditylenchus Filipjev*, 1936 (Nematoda: Anguinidae). Zootaxa.

[36] Zhao H.H., Liang C., Zhang Y., Duan F.M., Song W.W., Shi Q.Q., Huang W.K., Peng D.L. (2021). Research advances of biology in *Ditylenchus destructor* Thorne, 1945. Biotechnol. Bull..

[37] Liu W.Z., Liu Q.L., Ni X.M. (2003). Description of Stem Nematodes: *Ditylenchus destructor* Thorne, 1945. J. Laiyang Agric. Coll..

[38] Hooper D.J., Willmott S., Gooch P.S., Siddiqi M.R., Franklin M. (1973). C.I.H. Descriptions of Plant-Parasitic Nematodes.

[39] De Waele D., Jones B., Bolton C., Van den Berg E. (1989). *Ditylenchus destructor* in hulls and seeds of peanut. J. Nematol..

[40] Subbotin S.A., Madani M., Krall E., Sturhan D., Moens M. (2005). Molecular diagnostics, taxonomy, and phylogeny of the stem nematode *Ditylenchus dipsaci* species complex based on the sequences of the internal transcribed spacer-rDNA. Phytopathology.

[41] Wang J., Ji L., Huang G., Yang X., Lin M. (2007). Alignments of rDNA-ITS sequences and phylogeny of different geo-populations of *Ditylenchus destructor* in China. J. Agric. Univ. Hebei.

[42] Wang H.B., Mao J., Li R., Zhao G.D., Lin M.S. (2011). The pathogenicity study of hybrids populations of *Ditylenchus destructor* from different geographical origin. Acta Agric. Boreali-Sin..

[43] Wang C.L., Masler E.P., Rogers S.T. (2018). Responses of *Heterodera glycines* and *Meloidogyne incognita* infective juveniles to root tissues, root exudates, and root extracts from three plant species. Plant Dis..

[44] Liu C., Yang Y.W., Wang J.Z., Chang Q., Hong B., Zhang F. (2020). Homology analysis and identification of *Ditylenchus destructor* from different areas of Shaanxi province. Acta Agric. Boreali Occident. Sin..

